# Implication of species change of Nontuberculous Mycobacteria during or after treatment

**DOI:** 10.1186/s12890-017-0539-7

**Published:** 2017-12-20

**Authors:** Jong Sik Lee, Jong Hyuk Lee, Soon Ho Yoon, Taek Soo Kim, Moon-Woo Seong, Sung Koo Han, Jae-Joon Yim

**Affiliations:** 1 Division of Pulmonary and Critical Care Medicine, Department of Internal Medicine, Seoul National University College of Medicine, 101, Daehak-ro, Jongno-gu, Seoul, 03080 Republic of Korea; 2 Department of Radiology, Seoul National University College of Medicine, 101, Daehak-ro, Jongno-gu, Seoul, 03080 Republic of Korea; 3 Department of Laboratory Medicine, Seoul National University Hospital, 101, Daehak-ro, Jongno-gu, Seoul, 03080 Republic of Korea

**Keywords:** Nontuberculous mycobacteria species change, *Mycobacterium avium* Complex, *Mycobacterium abscessus* subspecies *abscessus*, Clarithromcycin resistance

## Abstract

**Background:**

Co-existence or subsequent isolation of multiple nontuberculous mycobacteria (NTM) species in same patient has been reported. However, clinical significance of these observations is unclear. The aim of this study was to determine clinical implications of changes of NTM species during or after treatment in patients with NTM lung disease.

**Methods:**

Patients with NTM lung disease, who experienced changes of NTM species during treatment or within 2 years of treatment completion between January 1, 2009 and December 31, 2015, were included in the analysis. Demographic, clinical, microbiological, and radiographic data were reviewed and analyzed.

**Results:**

During the study period, 473 patients were newly diagnosed with NTM lung disease. Treatment was started in 164 patients (34.6%). Among these 164 patients, 16 experienced changes of NTM species during or within 2 years of treatment completion. Seven showed changes from *M. avium* complex (MAC) to *M. abscessus* subspecies *abscessus* (MAA) and five patients displayed changes from *M. abscessus* subspecies *massiliense* (MAM) to MAC. With isolation of new NTM species, 6 out of 7 patients with change from MAC to MAA reported worsening of symptoms, whereas none of the five patients with change from MAM to MAC reported worsening of symptoms. All MAA isolated during or after treatment for MAC lung diseases showed inducible resistance to clarithromycin.

**Conclusions:**

Change of NTM species may occur during or after treatment for NTM lung disease. Especially, changes from MAC to MAA is accompanied by symptomatic and radiographic worsening as well as inducible resistance to clarithromycin.

## Background

Nontuberculous mycobacteria (NTM) are ubiquitous in environments including natural or treated water and soil. They have a relatively low pathogenicity but can cause lung disease in immunocompetent as well as immunocompromised hosts. [1, 2] Currently, approximately 150 NTM species have been identified. [3, 4] The incidence and prevalence of NTM lung disease are rising worldwide. [1, 5] The distribution of causative species of NTM lung disease varies according to country and region. In South Korea, *M. avium* complex (MAC) is the most common, comprising approximately 60%-70% of all cases, followed by *M. abscessus* complex, comprising approximately 20%-30%. [6–9].

Isolation of NTM different from initial NTM species in same patients has been reported. Co-culture of MAC was reported among 20% of patients with *M. abscessus* subspecies *abscessus* (MAA). [10] We also reported wide spectrum of NTM species changes including change of species, alternative isolation of two or three species, or simultaneous isolation of multiple species in same patients [11].

We have experienced several patients with NTM lung diseases in whom changes of NTM species was identified during or after treatment. However, the clinical significance this of NTM change has not yet been reported. Therefore, the aim of this study was to determine the clinical implications of changes of NTM species during or after treatment completion in patients with NTM lung disease.

## Methods

### Study population

Among patients treated for NTM lung disease at Seoul National University Hospital between January 1, 2009 and December 31, 2015, patients with changes of NTM species during treatment or within 2 years of treatment completion were included in the analysis. Demographic, clinical, microbiological, and radiographic data of the included patients were reviewed. This study was conducted in accordance with the amended Declaration of Helsinki. The study protocol was approved by the institutional review board of Seoul National University Hospital (IRB No: 1608-046-784), and written informed consent was obtained from all patients.

### Diagnosis of NTM lung disease and definition of newly isolated NTM species

Patients were diagnosed with NTM lung disease based on the diagnostic criteria of the American Thoracic Society (ATS)/Infectious Diseases Society of America (IDSA) guideline. [12] “Change of NTM species” were defined as disappearance of initially isolated NTM species and isolation of new species at least 2 times.

### Follow up and treatment

Patients with NTM lung disease underwent follow-ups every 3 to 6 months and treatment was offered in cases of significant radiographic progression (i.e. new cavity formation) or worsening respiratory symptoms (i.e. development of hemoptysis). Once treatment was initiated, patients visited the clinic every 4 to 8 weeks for physical examination, mycobacterial cultures of sputum, and radiographic evaluations. Treatment regimen was selected based on the ATS/IDSA guideline. [12] After treatment completion, patients underwent follow-ups every 3–6 months.

### Clinical and radiographic examination

Changes in respiratory symptoms were evaluated by the on-duty physician on every visit. If patients reported increased sputum production, worsened dyspnea, or development of hemoptysis, the symptoms were regarded as “worsened”. Likewise, “no change” of symptoms and “improved” symptoms were defined based on patients’ report.

**Fig. 1:**
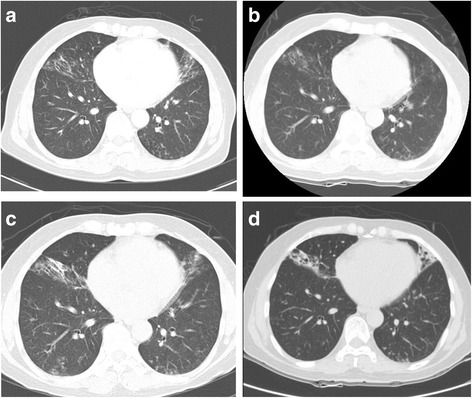
**a** Chest CT scan of a patient with symptomatic *Mycobacterium intracellulare* lung disease before initiation of treatment. The scan shows bronchiectasis, nodules, and reticular densities in the right middle lobe and lingular segment. Treatment started 4 weeks after checking this CT scan. **b** Chest CT scan at 6 months after the initiation of treatment showing substantial improvement. **c** Chest CT scan at 12 months after the completion of 18-month treatment for *M. intracellulare* lung disease. Radiographic lesions and symptoms worsened; bloody sputum was also noted. Four weeks after this CT scan was taken, *M. abscessus* subspecies *abscessus,* instead of *M. intracellulare,* was isolated. **d** Chest CT scan at 6 months after initial isolation of *M. abscessus* subspecies *abscessus*. The patient’s symptoms continued to worsen and sputum persistently tested positive for *M. abscessus* subspecies *abscessus*

Chest computed tomography (CT) was performed every 6 months during treatment and every 1–2 years after treatment completion. The severity of NTM lung diseases on CT was evaluated using a scoring system modified from a previously published one [13] by a board-certified radiologist (Fig. 1). The scoring system consists of severity, extent, and mucus plugging of bronchiectasis; severity and extent of cellular bronchiolitis; diameter, wall thickness, and extent of the cavity; nodules; and consolidation [13].

### Microbiological examination

At every visit, patients were requested to submit sputum samples for mycobacterial culture. Sputum were decontaminated with same volume of 4% sodium hydroxide (NaOH), homogenized, and concentrated by centrifugation at 3000×*g* for 20 min. The processed sediments were stained using the Ziehl-Neelsen method. [12] Concentrated specimen were cultured in 3% Ogawa medium to minimize possibility of contamination and observed weekly for 9 weeks after inoculation. Once cultured, *M. tuberculosis* and NTM were differentiated using Gen-Probe® method (Gen-Probe; San diego, CA, USA). [14] Following isolation of a suspected mycobacterial species, NTM was confirmed by analyzing the sequences of three genes: *16S rRNA*, *rpoB*, and *tuf*.

Antimycobacterial drug susceptibility tests were conducted at the Korean Institute of Tuberculosis by using broth microdilution. Minimum inhibitory concentrations (MICs) of antibiotics (amikacin, cefoxitin, ciprofloxacin, clarithromycin, imipenem, moxifloxacin, rifampicin, ethambutol, linezolid for MAC; and amikacin, cefoxitin, ciprofloxacin, clarithromycin, imipenem, moxifloxacin, and linezolid for *M. abscessus* complex) were determined according to the CLSI guidelines. [15] For MAC, isolates were considered as resistant if the MIC of clarithromycin was ≥32 μg/ml and as susceptible if the MIC of clarithromycin was ≤8 μg/ml. For *M. abscessus* complex, isolates were considered as resistant if the MIC of clarithromycin was ≥8 μg/ml and as susceptible if the MIC of clarithromycin was ≤2 μg/ml. Inducible resistance was considered if the MIC of clarithromycin was ≤2 μg/ml for 3 days and ≥8 μg/ml for 14 days.

### Statistical analysis

Data were summarized as medians with interquartile range (IQR) with non-normal distribution. We used repeated-measures data analysis with a Friedman model to test the significance of differences in CT scores. All statistical analyses were carried out using SPSS Statistics version 20 (IBM Corp, Chicago, IL, USA) and a *P* value < .05 was regarded as statistically significant.

## Results

### Characteristics of patients

During the study period, a total of 473 patients with NTM lung disease were diagnosed at Seoul National University Hospital. Of those, 164 patients started treatment for NTM lung disease. Among 164 patients with NTM lung disease, 54 patients (39 during treatment and 15 within 2 years of treatment completion) experienced isolation of another species of NTM. Among these 54 patients, 16 satisfied the definition of “change of NTM species”, 12 patients during treatment and in 4 patients within 2 years of treatment completion. The median number of isolation of new NTM species was 5.5 (IQR 3-7). The median age of these 16 patients was 69 years (IQR 61.7-73.5) and 12 (75%) were female. The median body mass index was 20.8 (IQR 19.2-21.4) (Table 1).

**Table 1:** Baseline characteristics of 16 patients with nontuberculous mycobacteria lung disease included for the analysis

Patients, No.	*N* = 16
Age, years, median (IQR)	69 (61.7-73.5)
Sex, female	12 (75.0%)
BMI, kg/m^2^, median (IQR)	20.8 (19.2-21.4)
Never smoker	14 (87.5%)
History of tuberculosis	6 (37.5%)
Underlying disease	
Connective tissue disease	4 (25.0%)
Diabetes	3 (18.7%)
Malignancy	1 (6.0%)
Respiratory symptoms	
Sputum	16 (100%)
Cough	13 (81.2%)
Dyspnea	4 (25.0%)
Hemoptysis	3 (18.7%)
General symptoms	
Weight loss	2 (12.5%)
Night sweating	1 (6.2%)
Laboratory findings, median (IQR)	
Leukocytes (×10^3^/μl)	6880 (5070-7542)
Hemoglobin (g/dl)	12.6 (11.9-13.3)
Cholesterol	168 (149-187.7)
Albumin	4 (3.8-4.4)
Creatinine	0.7 (0.6-0.9)

*BMI* body mass index, *IQR* interquartile range

### Changes of NTM species during or after treatment for NTM lung disease

Of 16 patients who showed change of NTM species during or after initial NTM lung disease treatment, 7 patients (43.8%) showed changes from MAC to MAA, 4 during treatment and 3 after treatment for MAC. Five patients (31.2%) displayed changes from *M. abscessus* subspecies *massiliense* (MAM) to MAC during treatment for MAM. The other four patients exhibited change from and to other NTM species, 3 during treatment and 1 after treatment for initial NTM (Table 2). Among 12 patients with change of NTM species during treatment, the median interval from starting treatment for initial NTM lung disease to new species isolation was 7.3 months (IQR 4.2-17.4). The median interval from treatment completion for initial NTM lung disease to isolation of a new species was median 15.6 months (IQR 14.9-16.4) among the other four patients.

**Table 2:** Changes of NTM Species During and After Treatment

	N = 16
*M. avium* complex → *M. abscessus* subspecies *abscessus*	7 (43.8%)
*M. intracellulare* → *M. abscessus* subspecies *abscessus*	4
*M. avium* → *M. abscessus* subspecies *abscessus*	3
*M. abscessus* subspecies *massiliense* → *M. avium complex*	5 (31.2%)
*M. abscessus* subspecies *massiliense → M. avium*	3
*M. abscessus* subspecies *massiliense → M. intracellulare*	2
Others	
*M. avium* → *M. fortuitum*	1 (6.2%)
*M. avium* → *M. intracellulare*	1 (6.2%)
*M. intracellulare* → *M. chimera*	1 (6.2%)
*M. abscessus* subspecies *massiliense* → *M. abscessus* subspecies *abscessus*	1 (6.2%)

### Changes of radiographic severities throughout treatment of initial NTM lung diseases and isolation of new NTM

Overall, the CT scores throughout the treatment course did not change. The median total CT scores at initiation of treatment for initial NTM, at 6–12 months after treatment, at isolation of a new NTM species, and at 6–12 months after isolation of a new NTM species was 13.2, 9.6, 12.9, and 13.0, respectively (*P* = .794) Likewise, CT scores before and after change of NTM species did not differ (median 12.9 vs 13.0, *P* = .763) (Table 3).

**Table 3:** Change of CT Scores Throughout Treatment of Initial NTM Lung Disease and Isolation of New NTM

	At initiation of treatment	At 6–12 months after treatment	At isolation of new NTM	At 6–12 months after isolation of new NTM	*P* Value
Bronchiectasis,					
Severity	1.7 (1-2)	1.8 (1.2-2)	2 (1.2-2)	1.7 (1.3-2)	.572
Extent	1 (1-2)	1 (1-2)	1 (1-2)	1 (1-2)	.733
Mucus plugging	1 (0-1)	0 (0-1)	1 (0-1)	1 (0-1)	.875
Cellular bronchiolitis					
Severity	1.8 (1.6-2.2)	1.7 (1.2-2)	2 (1.6-2)	1.8 (1.7-2)	.089
Extent	3 (1-3)	2.5 (1-3)	2 (1.5-3)	2 (1-3)	.112
Cavity					
Diameter (cm)	1 (0-2)	0 (0-1)	1 (0-1)	1 (0-2)	.245
Wall thickness (mm)	2 (0-2.5)	0 (0-2.1)	2 (0-2)	0.5 (0-2.4)	.978
Extent	1 (0-1)	0 (0-1)	1 (0-1)	1 (0-1)	1.000
Nodules	1 (0-1)	1 (0.8-1)	1 (1-1.5)	1 (0-1)	.572
Consolidation	0 (0-1)	0 (0-0.3)	0 (0-1)	1 (0-1)	.479
Total CT score	13.2 (7.2-17.6)	9.6 (6.4-14.4)	12.9 (9.0-14.8)	13 (8.0-14.6)	.794

Data are expressed as median (IQR). *CT* computed tomography, *NTM* nontuberculous mycobacteria

### Changes of respiratory symptoms with isolation of new NTM species

Six out of seven patients with change from MAC to MAA reported worsening of symptoms with isolation of new species. Conversely, no patient with change from MAM to MAC complained of worsening symptoms (Table 4).

**Table 4:** Symptomatic and Radiographic Changes, Clarithromycin Resistance, and Treatment for Newly Isolated NTM

No	NTM spices	Symptom changes at isolation of new NTM	Radiographic changes at isolation of new NTM	Clarithromycin resistance for initial NTM (MIC, μg/ml)	Clarithromycin resistance for new NTM(MIC, μg/ml)	Timing of new NTM isolation	Treatment for newly isolated NTM
*M. avium* complex → *M. abscessus* subspecies *abscessus*							
1	*M. intracellulare* → *M. abscessus* subspecies *abscessus*	Worsening	Worsening	Susceptible (1)	Inducible resistance (1, 64)	After treatment for initial NTM	Not started
2	*M. intracellulare* → *M. abscessus* subspecies *abscessus*	Worsening	Unchanged	Susceptible (2)	Inducible resistance (2, 64)	During treatment for initial NTM	^a^Started
`3	*M. intracellulare* → *M. abscessus* subspecies *abscessus*	Worsening	Worsening	Susceptible (2)	Inducible resistance (2, 8)	After treatment for initial NTM	Not started
4	*M. intracellulare* → *M. abscessus* subspecies *abscessus*	Worsening	Worsening	Susceptible (2)	Inducible resistance (0.5, 64)	After treatment for initial NTM	Not started
5	*M. avium* → *M. abscessus* subspecies *abscessus*	Unchanged	Unchanged	Susceptible (4)	Inducible resistance (0.5, 64)	During treatment for initial NTM	Not started
6	*M. avium* → *M. abscessus* subspecies *abscessus*	Worsening	Worsening	Susceptible (1)	Inducible resistance (1, 64)	During treatment for initial NTM	Continued on-going treatment
7	*M. avium* → *M. abscessus* subspecies *abscessus*	Worsening	Unchanged	Susceptible (0.5)	Inducible resistance (1, 64)	During treatment for initial NTM	Modified regimen and continued treatment
*M. abscessus* subspecies *massiliense* → *M. avium* complex							
8	*M. abscessus* subspecies *massiliense* → *M. intracellulare*	Unchanged	Unchanged	Susceptible (0.5)	N/A	During treatment for initial NTM	Modified regimen and continued treatment
9	*M. abscessus* subspecies *massiliense* → *M. intracellulare*	Unchanged	Worsening	Susceptible (0.5)	Susceptible (1)	During treatment for initial NTM	Modified regimen and continued treatment
10	*M. abscessus* subspecies *massiliense* → *M. avium*	Unchanged	Unchanged	Susceptible (0.5)	Resistance (64)	During treatment for initial NTM	Continued on-going treatment
11	*M. abscessus* subspecies *massiliense* → *M. avium*	Unchanged	Improving	Susceptible (0.5)	Susceptible (2)	During treatment for initial NTM	Modified regimen and continued treatment
12	*M. abscessus* subspecies *massiliense* → *M. avium*	Unchanged	Unchanged	N/A	N/A	During treatment for initial NTM	^a^Started
Others							
13	*M. avium* → *M. fortuitum*	Unchanged	Improving	Susceptible (0.5)	Inducible resistance (0.5, 16)	During treatment for initial NTM	Not started
14	*M. avium* → *M. intracellulare*	Worsening	Worsening	N/A	Susceptible (1)	After treatment for initial NTM	Started
15	*M. intracellulare* → *M. chimerae*	Improving	Worsening	Susceptible (1)	Susceptible (2)	During treatment for initial NTM	Continued on-going treatment
16	*M. abscessus* subspecies *massiliense* → *M. abscessus* subspecies *abscessus*	Unchanged	Worsening	Susceptible (0.5)	Inducible resistance (1, 16)	During treatment for initial NTM	Continued on-going treatment

*MIC* minimal inhibitory concentration, *N/A* not available, *NTM* nontuberculous mycobacteria

^a^Treatment for initial NTM lung disease was completed. Then, treatment for newly isolated NTM was initiated

### In vitro drug susceptibility to clarithromycin of newly isolated NTM species

All MAA isolated during or after the treatment for MAC lung diseases showed inducible resistance to clarithromycin. MAA isolated during treatment for MAM lung disease and *M. fortuitum* isolated during treatment for MAC lung diseases also showed inducible resistance to clarithromycin. One out of three MAC species isolated during or after treatment for MAM showed resistance to clarithromycin (Table 4). This patient refused to use intravenous drugs and took azithromycin only.

### Treatment for newly isolated NTM species

Among 12 patients in whom another NTM species were isolated during treatment for initial NTM lung disease, the same treatment regimens were continued in 4 patients while the regimens were modified for the newly isolated NTM in 4 patients. Despite of isolation of new NTM species, the other 4 patients continued to receive and completed treatments for initial NTM lung disease and were observed for a while; in 2 out of these patients, treatments for newly isolated NTM were started eventually. (Table 4).

Among 4 patients in whom new NTM species were isolated after the completion of treatment for initial NTM lung disease, only one started to receive treatment for newly isolated NTM. (Table 4).

## Discussion

In this study on patients with NTM lung diseases who received treatment, we identified several interesting findings. First, approximately 10% of patients with NTM lung disease exhibited changes to new NTM species during or within 2 years after treatment completion. Second, the most common pattern of NTM change species was from MAC to MAA. Third, change from MAC to MAA was associated with worsening of respiratory symptoms and radiographic lesions and most importantly with inducible resistance of clarithromycin.

Simultaneous or sequential isolation of several NTM species in the same patients has been reported. [10, 11, 16] In addition, repeated culture of different MAC strains in the same among patients with nodular bronchiectatic lung disease was also identified. [16] Through this study, we identified conversion of NTM species during or after treatment in 16 (9.8%) out of 164 patients treated for NTM lung diseases. The Majority of these patients (11 out of 16) experienced symptomatic and/or radiographic worsening with isolation of new NTM species. Similarly to the recent study [17], these could be regarded as development of new NTM lung disease.

In our study, conversion from lung disease by MAC to the caused by MAA, which is more difficult to treat, was most common than other conversion patterns. Furthermore, all MAA isolated from patients who had been treated for MAC lung disease showed inducible resistance to clarithromycin. Inducible resistance to clarithromycin involves a functional erm(41) gene, related to a T/C polymorphism at the 28th nucleotide. [18, 19] The rate of inducible resistance among these patients was very high compared with our recent study, which showed 55.1% of MAA isolated from patients in our hospital. [20] It could be explained that MAA with C28 sequevar, which do not show inducible resistance, might be eradicated by previous treatment with regimen including macrolide.

Previous studies reported that patients with different MAC strains usually have nodular bronchiectatic features rather than cavitary disease. [16, 21] Different strains might reside in different ectatic bronchi or nodules in same patients. Likewise, in our study, all 16 patients with conversion of NTM species during or after treatment had nodular bronchiectatic features. We speculate that these patients already had two NTM species in different ectatic bronchi or nodules at initial diagnosis of NTM lung disease. The number of initially detected major NTM species might be minimized with treatment while minor NTM species prevailed. For patients with conversion from MAC lung disease to MAA lung disease, treatment with macrolide, ethambutol, and rifampicin might reduce the number of MAC as well as MAA which is sensitive to clarithromycin, but MAA with inducible resistance could resist treatment and turn into the major NTM species in those patients.

This study has several limitations. First, the number of patients included in the analysis was small, although all patients included were treated at an institution that routinely diagnoses and treats a large number patient with NTM lung disease. To confirm our observations, a large-scale study enrolling patients from multiple hospitals is needed. Secondly, this study was performed retrospectively. Time points of requesting mycobacterial culture of sputum or chest CT scans were not controlled strictly. Additionally, crucial data, such as drug susceptibility test for the secondary isolated NTM species, were missing in some patients.

## Conclusions

NTM species changes could occur during or after treatment for NTM lung disease. Especially, changes from MAC to MAA were accompanied by symptomatic and radiographic worsening as well as inducible resistance to clarithromycin.

## Abbreviations

CT: Chest computed tomography
IQR: Interquartile range
MAA: *M. abscessus* subspecies *abscessus*
MAC: *M. avium* complex
MAM: *M. abscessus* subspecies *massiliense*
MICs: Minimum inhibitory concentrations
NTM: nontuberculous mycobacteria
